# Minerals in the Foods Eaten by Mountain Gorillas (*Gorilla beringei*)

**DOI:** 10.1371/journal.pone.0112117

**Published:** 2014-11-05

**Authors:** Emma C. Cancelliere, Nicole DeAngelis, John Bosco Nkurunungi, David Raubenheimer, Jessica M. Rothman

**Affiliations:** 1 Department of Anthropology, Graduate Center of the City University of New York, New York, New York, United States of America; 2 New York Consortium in Evolutionary Primatology, New York, New York, United States of America; 3 Department of Animal Science, Cornell University, Ithaca, New York, United States of America; 4 Department of Biology, Mbarara University of Science and Technology, Mbarara, Uganda; 5 Charles Perkins Centre, Faculty of Veterinary Sciences, School of Biological Sciences, The University of Sydney, Sydney, NSW, Australia; 6 Department of Anthropology, Hunter College of the City University of New York, New York City, New York, United States of America; Duke University School of Medicine, United States of America

## Abstract

Minerals are critical to an individual’s health and fitness, and yet little is known about mineral nutrition and requirements in free-ranging primates. We estimated the mineral content of foods consumed by mountain gorillas (*Gorilla beringei beringei*) in the Bwindi Impenetrable National Park, Uganda. Mountain gorillas acquire the majority of their minerals from herbaceous leaves, which constitute the bulk of their diet. However, less commonly eaten foods were sometimes found to be higher in specific minerals, suggesting their potential importance. A principal component analysis demonstrated little correlation among minerals in food items, which further suggests that mountain gorillas might increase dietary diversity to obtain a full complement of minerals in their diet. Future work is needed to examine the bioavailability of minerals to mountain gorillas in order to better understand their intake in relation to estimated needs and the consequences of suboptimal mineral balance in gorilla foods.

## Introduction

Minerals play a vital role in the growth and maintenance of animal tissues [1], including their involvement in maintaining structural components (e.g. magnesium [Mg], manganese [Mn], and phosphorus [P]), mediating enzymatic reactions (e.g. calcium [Ca], potassium [K], Mg, and zinc [Zn]), and maintaining acid-base balance (e.g. Ca) in the body [2], [3]. Mineral deficiency has both short- and long-term health costs, including compromised neuromuscular, gastrointestinal, cardiovascular, cognitive, or immune functioning [2]. This compromised functioning can impact fitness, with detrimental effects on fertility, growth, and mortality [3]. For example, short-term deficiencies in Ca can affect muscle function, nerve transmission, and blood clotting [4]. Prolonged Ca deficiency can cause chronic conditions including rickets and osteomalacia/osteoporosis in humans [5] as well as retard growth and cause abnormalities to bone and teeth [6]. Despite its importance, our understanding of the mineral intake and requirements of wild primates is limited [2], [7]. Few studies have investigated the dietary minerals of primates [7], [8], [9], [10], [11], [12], [13], [14], and particularly of apes [15], [16], [17], [18], [19].

The environmental availability of minerals in primate habitats has been suggested as a potential limiting factor to population growth in redtail monkeys (*Cercopithecus ascanius*) [20], and movement patterns of black and white colobus (*Colobus guereza*) are dictated to some extent by the sodium found in *Eucalyptus* trees [21]. Gorillas (*Gorilla gorilla*) in swampy areas select foods that are rich in Ca and Na [15]. Mineral acquisition strategies also vary based on ability to utilize consumed minerals, and the bioavailability of minerals within food items; minerals first have to be found in suitable foods, and then must be available for digestion and absorption. Given the variety of dietary types and digestive systems within the primate order, taxa may differ greatly in their mineral requirements and strategies to acquire mineral nutrients. For example, howler monkeys rely heavily on figs in their diets, a fruit that is high in Ca [9], and thus may not seek to otherwise supplement their diet with Ca, and colobines that host foregut microbes may have a lower need for certain minerals (similar to ruminants) [22], [23]. When staple foods do not provide sufficient minerals, primates can meet their mineral needs by supplementing their typical diet. Several distinctive and unusual feeding behaviors have been suggested to serve a mineral acquiring function, including geophagy (e.g. in chimpanzees [24], *Pithecia* [25], and *Macaca* [26]), consumption of wood (e.g. *Ateles* [8], mountain gorillas [17], chimpanzees [18]), insectivory [27], or consumption of liquids like urine and swampy waters (e.g. *Procolobus* monkeys [7], [28]).

Bwindi mountain gorillas (*Gorilla beringei beringei*) live in montane forests characterized by high-protein herbaceous plants, with seasonal availability of fruit [29], [30], while the mountain gorillas in the neighboring Virunga Volcanoes are almost exclusively folivorous as fruiting trees are not available in their high altitude habitat [31], [32]. In comparison, western lowland gorillas (*Gorilla gorilla gorilla*) are more frugivorous than the Bwindi population, consuming fruit almost daily [32]. Their respective food availability may have implications for the mineral compositions of their diets.

The diet of the Bwindi gorilla has been previously described [30], [33], [34], [35] with some reference to mineral nutrition. Their diet is relatively diverse, comprising 148 food items from 107 species of plant [30]. Nevertheless, over 90% of the Bwindi mountain gorilla diet consists of 17 staple foods, while the remaining food items each contribute less than 1% to the total diet [30]. Some of the less commonly eaten foods may be important sources of minerals; in particular decaying wood has been shown to provide the majority of sodium in Bwindi gorilla diets [17]. A study in Bwindi gorillas addressing nutrition across age/sex classes found that mineral intake varied [35]. However, in all age/sex classes, mineral intake was consistent with or exceeded adequate daily concentrations recommended for Hominidae by the National Research Council (NRC) [2], with exceptions being Zn, Na, and P [35].

We characterized the mineral compositions of mountain gorilla (*G. b. beringei*) food items to better understand potential mineral acquisition strategies. We predicted that mountain gorillas would gain most of their minerals from the herbaceous leaves in their diet, and fruits would be a relatively poor source of minerals compared to leaves [11].

We also examined the ratios of minerals in food items known to have interactive effects in the diets of mammals [3]. Interactions between co-occurring minerals can profoundly impact their bioavailability, such that excesses or deficits in one mineral can inhibit the absorption of another [36]. For example, when P is excessively high in relation to Ca, the body will stop absorbing Ca and the mineral may be actively removed from the blood plasma [37]. If minerals are not properly balanced (i.e., consumed in specific proportion relative to other minerals, in order to be used optimally by biological tissues [3]), mineral deficiency may occur at a cellular or tissue level, despite the consumption of a sufficient amount of each mineral in isolation.

Finally, we compared the minerals in mountain gorilla foods to minerals in the diets eaten by the more frugivorous western lowland gorilla (*Gorilla gorilla*) [15], [16], and the minerals in of leaves eaten by a diversity of primates.

## Methods

### Study site and animals

Bwindi Impenetrable National Park (BINP) is located between 0°53′ and 1°08′S, 29°35′ and 29°50′E in southwestern Uganda, and our research was conducted at the Institute of Tropical Forest Conservation in Ruhija sector. The landscape is characterized by rugged mountainous rainforest, with steep hills and narrow valleys. BINP contains one group of mountain gorillas specifically habituated for research, the Kyagurilo group [35]. Details of the Kyagurilo group are outlined in previous publications [33], [34], [35].

Researchers are permitted to carry out observations for a maximum of four hours a day, in order to minimize both disturbance and disease risk to the gorillas. Typically, these four hours occurred between 0830 to 1500 hours for this study, but they varied throughout the day.

### Plant collection and nutritional analysis

As outlined previously [33], [34], [35], food items consumed by the gorillas during observation were collected within the same week they were consumed. When possible, samples were taken from the exact plant consumed, or from directly adjacent plants of the same species. Food items were processed in a manner similar to how the gorillas processed the food (i.e. if only certain parts of the plant were eaten, only those plant parts were processed for analysis). For mineral analysis, 103 plants were analyzed and one rock seen to be ingested by the gorillas.

Plants were weighed immediately after collection using a portable balance and then the samples were dried at ≤22°C at the field station until a constant weight was achieved. Dried samples were ground at Makerere University in Uganda, using a Wiley Mill with a 1-mm screen. Mineral content (sodium [Na], Ca, P, Mg, K, iron [Fe], Zn, copper [Cu], and Mn) was determined using a Thermo Jarrell Ash IRIS Advantage Inductively Coupled Plasma Radial Spectrometer at Dairy One Forage Laboratory, Ithaca, New York, USA. We present mineral content on a dry matter basis.

### Statistical analysis

Samples were grouped into one of six plant part categories: bark, fruit, herbaceous leaves, tree leaves, pith/stem (including both pith, the outer green peel on herbs, and stem material), and root. Bark was defined as the outer bark of trees and twigs (woody material). Mineral compositions across plant parts were compared using nonparametric Kruskal-Wallis tests, with multiple comparisons conducted based on Dwass, Steel, & Critchlow-Fliger pairwise rankings, with an *a priori* alpha level of 0.05 [38]. These analyses were conducted using StatsDirect. We also conducted a principal component analysis (PCA) in R to assess potential underlying trends in plant part categories and mineral content of individual samples [39].

The ratios of minerals in foods were calculated for select mineral pairs (Ca:P, Ca:Na, Ca:K, Ca:Mg, Na:Mg, Zn:Cu, and Fe:Cu) as per the NRC’s nutritional guidelines for non-human primates [2], to enable comparisons to recommended ratios. Ratios for food items were calculated by weighting mineral contents for the most commonly consumed gorilla food items (accounting for 80% of the total diet) [40] by the percent intake of each food item [40]. Mineral ratios then were presented as averages based on total dietary intake [40]. The mineral content of decaying wood was previously reported [17], and was therefore not included in summary figures.

The NRC non-human primate guidelines for Hominidae, based on recommended human values (Table 11-1 of NRC [2]), were used as a standard for comparison to observed mineral intake in mountain gorillas [2].

## Results

### Mineral content of food items

**Figure 1:**
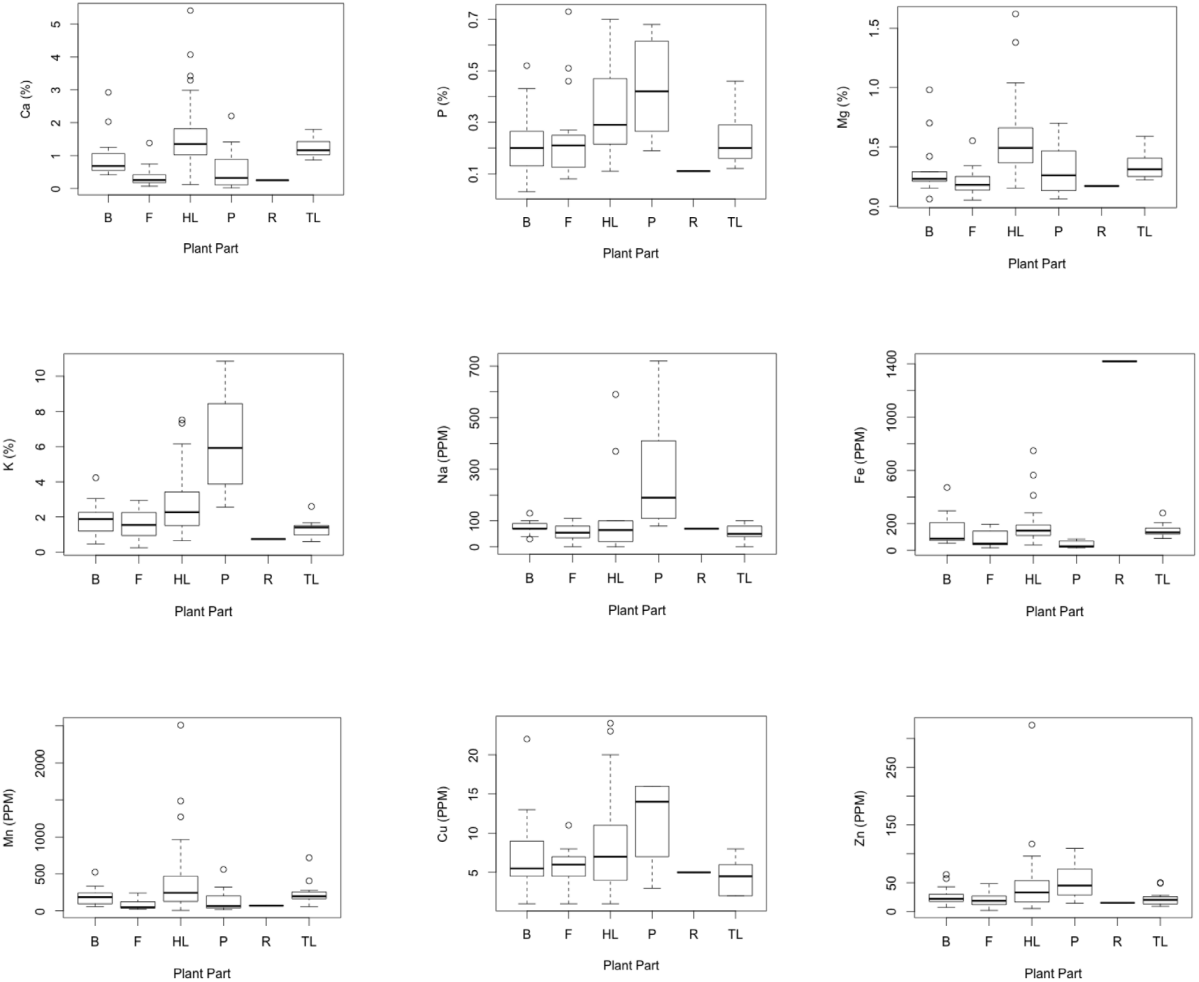
Comparisons of mean mineral composition in gorilla food items at Bwindi Impenetrable National Park.

Plant parts differed in concentrations of Ca (*H = *36.56, *P<*0.001), P (*H = *14.99, *P = *0.01*)*, Mg (*H = *31.85, *P<*0.001), K (*H = *27.15, *P<*0.001), Na (*H = *15.26, *P = *0.01), Zn (*H = *12.24, *P = *0.03), Fe (*H = *29.15, *P<*0.001), and Mn (*H = *23.64, *P<*0.001), but not in concentrations of Cu (*Figure 1*). Roots were higher than all other plant parts in Fe (*Table 1*), and a single ingested rock sample analyzed from the site was also very high in Fe (2,520 PPM). Pith/stem was higher than bark, fruit, herbaceous leaves, and tree leaves in K, and had the highest mean concentrations for P, Zn, and Cu. Herbaceous leaves had the highest mean concentrations of Ca, Mg, and Mn, and there were differences between herbaceous leaves and fruit for Ca, herbaceous leaves and fruit for Mn, and herbaceous leaves, bark, and fruit in Mg (*Table 1*).

**Table 1:** Mean mineral concentrations in food items eaten by mountain gorillas (Gorilla beringei) in Bwindi Impenetrable National Park.

Part	N	Ca (%)	P (%)	Mg (%)	K (%)	Na(PPM)	Fe(PPM)	Zn(PPM)	Cu(PPM)	Mn(PPM)
**Bark**	13	**0.96a**	**0.22a**	**0.31ab**	**1.89ab**	**75a**	**149abc**	**26a**	**7a**	**194a**
**SD**		0.68	0.14	0.23	0.95	25	115	16	5	122
**Range**		0.41–2.92	0.03–0.52	0.06–0.98	0.47–4.23	30–130	52–472	7–64	1–22	58–522
**Fruit**	16	**0.36b**	**0.25a**	**0.21a**	**1.59ab**	**55a**	**81ac**	**20a**	**5a**	**79b**
**SD**		0.33	0.18	0.12	0.79	29	58	11	2	63
**Range**		0.07–1.38	0.08–0.73	0.05–0.55	0.25–2.94	20–110	19–195	2–48	1–11	22–240
**Herb leaves**	27	**1.62c**	**0.34a**	**0.56c**	**2.71a**	**81a**	**181b**	**44a**	**8a**	**411a**
**SD**		1.08	0.17	0.31	1.72	106	136	54	6	504
**Range**		0.12–5.41	0.11–0.7	0.15–1.62	0.65–7.51	20–590	39–748	5–323	1–24	7–2511
**Tree leaves**	20	**1.25ac**	**0.24a**	**0.35bc**	**1.35b**	**56a**	**152ab**	**23a**	**4a**	**246a**
**SD**		0.29	0.11	0.12	0.53	32	52	14	2	181
**Range**		0.87–1.8	0.12–0.46	0.25–1.67	0.6–2.6	40–100	90–280	9–50	2–8	55–718
**Pith**	7	**0.65abc**	**0.44a**	**0.32abc**	**6.28c**	**290b**	**45c**	**53a**	**11a**	**159ab**
**SD**		0.83	0.20	0.23	3.25	236	29	36	5	205
**Range**		0.02–2.2	0.19–0.68	0.06–0.48	2.56–10.85	80–720	19–86	14–109	3–16	17–560
**Root**	1	**0.25**	**0.17**	**0.11**	**0.75**	**1420**	**15**	**5**	**70**	**70**
**SD**		-	-	-	-	-	-	-	-	-
**Range**		-	-	-	-	-	-	-	-	-
**Silverback male daily mineral** **intake (mg per unit M) ** [35]		392	67	116	612	0.05	2.42	0.64	0.18	8.30
**Female daily mineral intake** **(mg per unit M) ** [35]		733	131	225	1013	0.06	4.34	1.34	0.33	16.2
**Juvenile daily mineral intake** **(mg per unit M) ** [35]		931	192	292	1597	0.08	7.52	2.09	0.50	21.5

Differences in mineral concentrations between plant parts (P<0.005) are indicated by letter differences (per column). Shared letters indicate no significant differences in mineral concentration (per column).^1^

1 Food items include bark, fruit, herbaceous leaves, tree leaves, pith/stem (including both pith, the outer green peel on herbs, and stem material), and root. Bark was defined as the outer bark of trees and twigs (woody material). Ca = calcium, P = phosphorus, Mg = magnesium, K = potassium, Na = sodium, Fe = iron, Zn = zinc, Cu = copper, Mn = manganese. PPM = Parts per million, % = Percentage on a dry matter basis. All pairwise comparisons are based on Dwass, Steel, & Critchlow-Fliger pairwise rankings.

The mineral ratios of gorilla food items analyzed rarely met ratios suggested by the NRC [2] (*Table 2*). Na:K ratios, Na:Mg ratios, and Fe:Cu ratios were consistently outside of the recommended range.

**Table 2:** Mean ratios of minerals in staple foods eaten by Bwindi Mountain gorillas, weighted by daily intake (measured in g) [40].

	% DailyIntake [40]	Ca:P	Ca:Na	Ca:K	Ca:Mg	Na:Mg	Zn:Cu	Fe:Cu
Herbaceous leaves	61%	4.36	703.46	0.66	2.14	<0.001	3.24	9.81
Fruit	13%	0.16	2.40	0.02	0.19	<0.001	0.21	1.07
Pith	6%	0.19	1.37	0.01	0.14	0.01	0.23	0.14
Ratio for allstaple foods [40]		2.81	427.01	0.41	1.43	0.01	2.17	6.19
Recommendedratio [2], [61]	–	1.57	0.67	0.21	2.97	10.50	8.89	1.57

Staple foods considered accounted for 80% of total diet [40].

### Mineral diversity and associations between minerals

**Figure 2:**
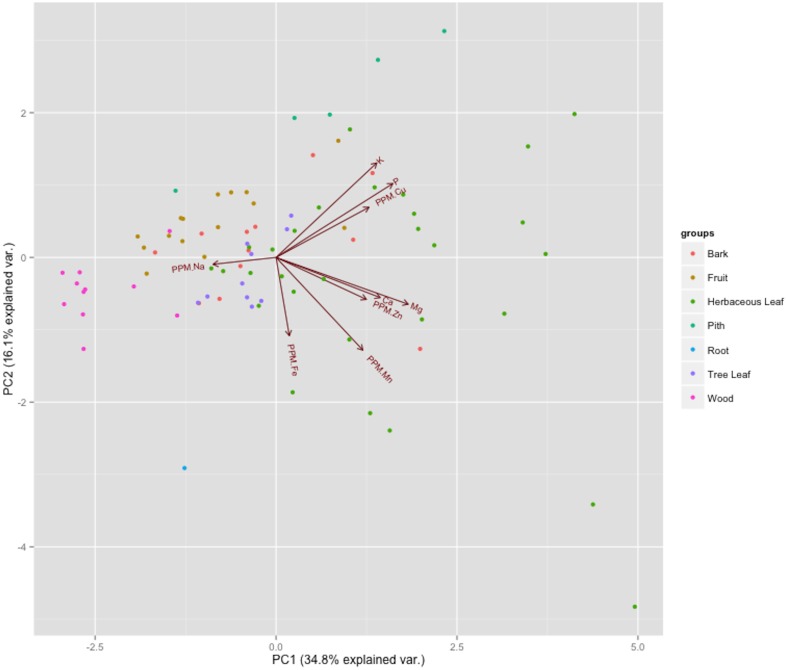
Biplot showing the first two loadings of the principal component analysis of all mineral values in samples analyzed. Food items grouped by color.

The PCA showed similar patterns of association between minerals, with Ca, Mg, and Zn grouping most closely together according to the first and second principal components (*Figure 2*). Food part categories did not cluster in multivariate space, with the first and second principal components together explaining only 50.8% of the variation in mineral quantities between plant parts (34.8% and 16.1% respectively) (*Table 3*). A plot of the principal components indicates that subsequent components each explain a fairly even, consistent proportion of variation in data. Thus, no single underlying association seems to have greatly influenced mineral presence or concentrations in plant parts or samples; rather, individual foods and groups of food items are highly variable in their mineral profiles and scatter relatively randomly in multivariate space.

**Table 3:** Loadings of the first two components of a principal component analysis of associations between minerals.

	Component 1	Component 2
**Ca**	0.37	–0.21
**P**	0.41	0.39
**Mg**	0.47	–0.24
**K**	0.35	0.49
**Na**	–0.22	–0.04
**Fe**	0.05	–0.41
**Zn**	0.32	–0.22
**Cu**	0.33	0.26
**Mn**	0.31	–0.48

### Comparison to mineral nutrition in western lowland gorilla foods

Mineral compositions of Bwindi gorilla food items are generally similar to those in western lowland gorilla food items (*Table 4*). However, Cu in western lowland gorilla fruits, leaves, and shoots was higher than in comparable foods eaten by Bwindi gorillas while leaves at Bwindi were lower in Na, and higher in P and Mg.

**Table 4:** Comparison of mineral composition of food items between *Gorilla beringei* and *Gorilla gorilla* [16] 1.

			*G. beringei*	*G. gorilla*	*G. beringei*	*G. gorilla*	*G. beringei*	*G. gorilla*
Part	n	n	Ca (%)		P (%)		Mg (%)	
**Leaf**	**47**	**8**	**1.53**±0.96	1.34±0.97	*0.32±0.16 (*p = 0.022)*	0.18±0.07	*0.51±0.29 (*p = 0.038)*	0.29±0.153
**Bark**	**13**	**2**	**1.02**±0.72	1.23±0.11	**0.22**±0.14	0.07±0.02	**0.34**±0.23	0.12±0.113
**Fruit**	**16**	**7**	**0.36**±0.33	0.35±0.39	**0.25**±0.17	0.18±0.11	**0.21**±0.12	0.16±0.069
**Shoot**	**2**	**5**	**0.07**±0.07	0.39±0.16	**0.42**±0.29	0.22±0.08	**0.16**±0.14	0.26±0.089
**Root**	**1**	**1**	**0.25**	0.11	**0.11**	0.07	**0.17**	0.04
**Part**	**n**	**n**	**K (%)**		**Fe (PPM)**		**Zn (PPM)**	
**Leaf**	**47**	**8**	**2.39**±1.62	1.68±0.770	**175.02**±122.31	274±217.881	**39.87**±48.46	34.88±26.292
**Bark**	**13**	**2**	**2.01**±0.96	1.65±0.919	**162.23**±119.29	105.5±77.074	**27.92**±16.81	10.5±3.535
**Fruit**	**16**	**7**	**1.59**±0.79	1.81±1.009	**81.38**±58.63	206±404.691	**20.13**±11.49	52.75±101.66
**Shoot**	**2**	**5**	**3.34**±1.09	3.02±1.207	**43.5**±20.50	93.40±16.890	**38**±16.97	48±16.093
**Root**	**1**	**1**	**0.75**	1	**1420**	37	**15**	14
**Part**	**n**	**n**	**Cu (PPM)**		**Na (PPM)**		**Mn (PPM)**	
**Leaf**	**47**	**8**	**7.74**±5.97	*14.13±6.010 (*p = 0.012)*	**75.75**±95.04	*178.75±194.546 (*p = 0.021)*	**372.6**±453.32	284±136.32
**Bark**	**13**	**2**	**8.2**±5.69	6±0	**76.15**±26.94	85±49.497	**208.39**±125.41	155±49.497
**Fruit**	**16**	**7**	**5.53**±2.47	*13.43±9.288 (*p = 0.008)*	**55**±29.44	*119.38±160.14 (*p = 0.013)*	**79.75**±63.29	140±158.19
**Shoot**	**2**	**5**	**5**±2.82	14.00±5.612	**85**±7.071	155±133.32	**47**±42.43	552±371.44
**Root**	**1**	**1**	**5**	4	**70**	70	**70**	0

1 *, denotes significantly higher values (*P<0.05*) as determined by Mann-Whitney U tests of significance. Samples with an n<2 were excluded from analysis.

## Discussion

### Mineral Composition

Our study suggests that food items consumed by Bwindi mountain gorillas differ substantially in their mineral profiles both between and within plant part categories. Roots were higher in Fe compared to all other plant parts, pith/stem was higher in K compared to bark, fruit, herbaceous leaves, and tree leaves, and herbaceous leaves had the highest mean concentrations of Ca, Mg, and Mn compared to all other plant/plant parts tested.

Conversely, certain food items were found to be very low in their mineral concentrations when compared to other food items. The fruits analyzed in this study were low in their mineral content. The low mineral quality of fruits is well documented [11] (an exception being figs, which act as an important source of Ca for many primate species [9], [41]). The mineral content of mountain gorilla food items was similar to the foods consumed by western lowland gorillas in Cameroon ([16]; *Table 4*). Herbaceous leaves, an important food item for mountain gorillas [30], were equal or higher in Ca, K, Mn, and P than tree and herbaceous leaves consumed by other free-ranging primates at sites across Africa, Asia, and the Americas (*Table 5*).

**Table 5:** Mineral content of leaves consumed by gorillas at BINP compared to leaves consumed by wild primates at other research sites.

Species	Location	n	% Ca	% P	% Mg	% K	% Na	PPM Fe	PPM Zn	PPM Cu	PPM Mn
***Gorilla beringei*** **(this study)**	BINP	47	1.53	0.32	0.513	2.387	0.007	175.021	39.87	7.743	372.6
***Nasalis*** ***larvatus*** [14]	Indonesia	17	0.634	0.069	0.312	0.726	0.003	35.2	13.9	–	122
***Alouatta*** ***nigra*** [13]	Belize	33	1.305	0.27	0.52	1.83	0.032	95.56	30.19	15.025	–
***Gorilla*** ***gorilla*** [15]	Congo	-	2.63	0.77	0.58	0.307	0.218	–	–	–	–
***Macaca sylvanus,*** ***Lemur catta*** [10]	Georgia,USA	37	0.73	0.15	0.32	–	0.04	84.2	24.1	5.6	114.4
***Gorilla*** ***gorilla*** [16]	Cameroon	8	1.341	0.181	0.286	1.675	0.018	274	34.88	14.13	284
***Pan troglodytes*** [18]	Budongo,Uganda	4	0.597	0.132	0.268	1.624	–	84.25	103.5	–	93.25
***Colobus guereza,*** ***Procolobus tephrosceles*** [7]	Kibale,Uganda	106	1.02	–	0.28	1.64	–	146.45	25.85	9.95	139.6

### Dietary diversity, food selection, and mineral composition

To obtain sufficient quantities of all minerals, it is important for gorillas to consume a wide range of different food items. As suggested by Milton [42], this strategy of selecting a diversity of food items, each item high in particular minerals, may allow primates to achieve optimal micronutrient nutrition in habitats that are typically mineral-poor [7], [8], [9], [10]. The relationship between dietary diversity and likelihood of obtaining an adequate complement of nutrients has been observed across animals in general [43], [43], [44,45]. In humans, increases in dietary diversity can contribute to longer life expectancy and lower infant mortality [46], [47], and dietary diversity is often used as an indicator of nutritional adequacy [48].

The PCA results support the idea that high dietary diversity allows for the acquisition of a full complement of minerals. The first two principal components were driven by weak or moderate associations between minerals [39], indicating that minerals do not associate strongly along an underlying gradient or set of parameters. The overlap in plant parts and the high variation within groups together indicate that plant part does not indicate any generality to the mineral composition of a food item.

Much debate exists in the current literature as to the importance of minerals in driving food selection [7], [9], [10], [15], [17], [18], [28], [42], [49]. The consumption of certain foods that are low in macronutrients, like wood and roots, is likely explainable by their mineral composition. Wood consumption in mountain gorillas has been previously related to its high Na content [17] and gorillas select stumps that are high in sodium, a behavior observed in other primates as well [8], [18]. Nevertheless, it remains unclear as to whether mountain gorillas are selecting specifically for mineral content in their food. Future studies should investigate mineral temporal and spatial availability in relation to consumption.

Although pith might be selected for water content, or its high level of easily digestible sugars or hemicellulose [50], it is possible that pith consumption is at least in part driven by its high K composition. The piths consumed by Bwindi mountain gorillas contain large percentages of water (up to 96% water content) [33], high levels of fiber, and low levels of crude protein [34]. Although K deficiencies are rare due to its abundance in plants [2], selection for K has been noted in folivorous mammals. For example, the folivorous Brazilian rodent *Kerodon rupestris* has been shown to select low quality foods (low macronutrient content) in order to meet daily minimum K requirements, even during periods of food resource limitation [51]. However, given that mountain gorillas consume a much higher level of K than minimally required [35], alternative explanations are likely required to account for this behavior in mountain gorillas.

### Mineral Ratios

The ratios of minerals found in individual gorilla food items rarely met acceptable targets as per the NRC’s guidelines for nonhuman primates [2]. Minerals consumed in excess or deficit relative to the proportion of other minerals may have compromised bioavailability, as a result of mineral interactions within the body [3], [36]. Such unbalanced mineral ratios can have adverse health effects. The relationships among Ca, P, and Mg in particular have strong implications for health; for example, captive primates fed diets unbalanced in these minerals can develop a series of skeletal deformities throughout their lifespan [37]. Within this group of minerals, the proportion of P in relation to Ca is especially tightly interwoven [52]. Calcium:Phosphorus ratios in gorilla food items were generally higher than the NRC-recommended ratio, even when considered in the context of dietary intake [35], [40].

Unbalanced ratios occur in other dietary minerals, as well. When considered within the context of dietary intake per unit body mass, the Ca:Mg ratio for silverback males is lower than the ratio recommended for good health in primates, whereas the Ca:K ratio of total dietary intake lies above the recommended ratio [35], [40]. In dairy cattle it has been suggested that high levels of K interfere with Ca absorption and lead to higher incidence of milk fever (a hypocalcemic state that leads to appetite loss, weakness, and heart failure), though this interaction may be unique to foregut fermenters [53]. Both mountain gorilla foods and their diets overall have a higher Ca:K ratio than recommended, but the direct implications of this ratio are unknown. It should also be noted that published mineral requirements and ratios might be conservative because primates vary in body size, physiology and digestion, and foods vary in bioavailability [54]. While we compared gorilla foods to recommended Hominidae requirements, the recommendations for adequate concentrations of minerals for nonhuman primates may be higher than what primates actually require. For example, the NRC suggests that primates need to consume a diet of 0.25% Na on a dry matter basis, but the diets of most wild primates are much lower [7], [35], indicating that primates are able to survive on lower dietary concentrations of Na.

Given that the mineral ratios do not always meet recommended target ratios when considered within the context of overall diet, the bioavailability of minerals in gorilla foods becomes important in understanding the implication of mineral ratios in mountain gorilla diets. The ratio of Ca to P ratio in gorilla feces is 4.04 (Rothman, *unpublished data*), higher than the ratio averaged across all staple foods suggested by this study (2.81) but lower than that of the major dietary component, herbaceous leaves (4.36). The Ca:P ratio in feces, however, is lower than that observed in the daily diets of gorilla females (5.59), silverback males (5.85), and juveniles (4.85) [35], which is considerably higher than the recommended ideal ratio for humans (1.57, *Table 2*). This suggests that the high levels of Ca in the diet might inhibit the absorption of P, which occurs when Ca is consumed in excessive amounts [33], [52].

In addition to mineral interactions, the bioavailability of minerals can also be affected by plant physiology. Roots, for example, carry high percentages of the minerals abundant in the surrounding soil [55], and the single rock sample ingested by an individual at the site was found to be exceedingly high in Fe (2,520 PPM). The Fe in plant tissues tends to be predominantly unavailable for digestion, as it is usually bound to organic compounds in the plant structure that may past through animal digestive tracts [56]. While little is known about the use and uptake of Fe in roots [56], the availability of Fe has been tested in legumes, where levels of Fe-binding polyphenols and the presence of phytate (an inhibitor of Fe absorption) render most Fe unusable to animals, despite the high overall content of Fe found in these plant structures [57]. Understanding patterns of bioavailability in Fe is especially crucial in primates, as captive primates have been shown to be highly vulnerable to hemosiderosis (iron overload) as a result of overconsumption of Fe [11], [58].

### Future Directions

Moving forward, it is critical to better understand the biological availability of minerals to gorillas, and how the mineral composition of plants relates to dietary selection and mineral nutrient acquisition. Information on bioavailability in primates is scarce [41], [59], but non-invasive methods to estimate bioavailability are available [60]. Employing these methods would allow researchers to make more accurate statements pertaining to mineral ratios, potential mineral targets, and the importance of minerals as a deciding factor in dietary choices. Lastly, understanding mineral composition within the context of dietary contribution would allow us to further explore the hypothesis that increasing dietary diversity and supplementation with low-macronutrient, high-mineral foods optimizes mineral intakes in mountain gorillas.
